# A Escolha da Prótese Valvar para o Sucesso da Gravidez. A “Ponta do Iceberg” para uma Doença de Evolução Complexa

**DOI:** 10.36660/abc.20240163

**Published:** 2025-02-20

**Authors:** Walkiria Samuel Avila, Daniel Vinicius Rodrigues Pinto, Jessica Sol Brugnara, Marilia Moro, Talita Carla Stratti Moreira, Isabelle Borges, Nana Miura, Flávio Tarasoutchi

**Affiliations:** 1 Hospital das Clínicas Faculdade de Medicina Universidade de São Paulo São Paulo SP Brasil Instituto do Coração do Hospital das Clínicas da Faculdade de Medicina da Universidade de São Paulo, São Paulo, SP – Brasil

**Keywords:** Próteses Valvares Cardíacas, Gravidez, Morte materna, Anticoagulantes

## Abstract

**Fundamento:**

Escolher prótese valvar no planejamento de gravidez, ainda é controverso. Tanto as próteses biológicas quanto as mecânicas têm limitações para o sucesso da gravidez.

**Objetivos:**

Estudar o índice de sucesso da gravidez após o implante de prótese valvar, e identificar as variáveis relacionadas aos desfechos maternos.

**Métodos:**

Estudo prospectivo de 78 gestantes com prótese de pericárdio bovino (Grupo PB) e 50 com prótese mecânica (Grupo PM) quanto ao índice de sucesso da gravidez, considerado na ausência de complicações cardíacas, obstétricas e/ou fetais.

**Resultados:**

O sucesso foi alcançado em 64 (50,0%) pacientes, e não foi diferente entre os grupos (PB 56,4% vs. PM 40,0% - p=0,103). O grupo PB teve maior frequência de eventos cardíacos e de disfunção de prótese (43,6% vs. 16,0% p<0,001; 26,9% vs. 2,0% p<0,001), contudo perdas fetais (14,1% vs. 24,0% p=0,165) e complicações obstétricas (28,2% vs. 42% p=0,127) não foram diferentes entre os grupos. A insuficiência cardíaca (odds ratio 8,5; CI 95% [1,4; 50,7]; p=0,019), fibrilação atrial (odds ratio 16,7; CI 95% [5,7; 49,1]; p<0,001) e disfunção da prótese biológica (odds ratio 12,6; CI 95% [3,0; 52,7]; p=0,001) anteriores à gestação foram as variáveis de predição de insucesso da gravidez.

**Conclusões:**

Mulheres com próteses valvares tiveram baixo êxito materno-fetal decorrentes de fatores complicadores da doença valvar, da limitada sobrevida estrutural das próteses biológicas e da inexistência de anticoagulantes que assegurem uma gestação. A escolha da prótese valvar para a gravidez, não deve ser isolada, mas deve fazer parte de uma doença cardíaca de evolução complexa.

## Graphical abstract

**Figura Central:**
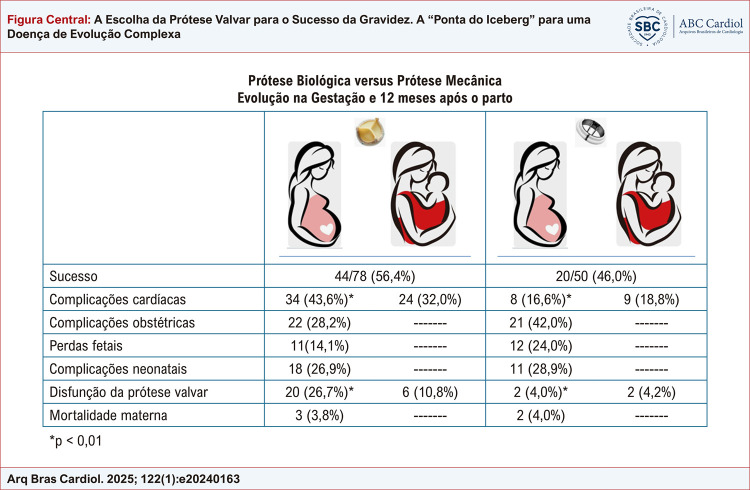
A Escolha da Prótese Valvar para o Sucesso da Gravidez. A “Ponta do Iceberg” para uma Doença de Evolução Complexa

## Introdução

O implante de prótese valvar tem possibilitado o desenvolvimento da gestação em pacientes com lesões cardíacas estruturais graves, consequentes a doença valvar de etiologias, reumática e congênita.

A escolha do substituto valvar para mulheres em idade reprodutiva deve considerar que ambas as próteses, biológica (PB) e mecânica (PM), apresentam particularidades que determinam riscos materno e fetal, tendo em vista uma futura gravidez.

A durabilidade limitada da PB em mulheres jovens e a necessidade de reoperação são os grandes entraves para essa escolha na faixa etária reprodutiva. Em contrapartida, a natureza trombogênica da PM e o estado de hipercoagulabilidade do ciclo gravídico-puerperal, requerem o uso de anticoagulantes permanente e eficaz, a despeito dos sérios efeitos adversos para a gravidez.

As limitações inerentes à maioria dos estudos sobre próteses valvares e gestação, são relacionadas ao seu caráter retrospectivo, à casuística heterogênea e às imperfeições na metodologia, o que impedem a acurácia ideal em validar conclusões.^1-5^

Essas imprecisões, motivaram este estudo sobre gestações em mulheres após o implante de prótese valvar, que seguiram critérios rígidos de padronização do substituto valvar, da conduta assistencial prévia a gestação e até 12 meses após o parto, e do protocolo para o uso de anticoagulantes.

O objetivo principal do estudo, foi avaliar a gestação e até 12 meses após o parto, mulheres após o implante de prótese valvar e comparar a evolução, entre PB e PM. Os objetivos secundários foram: analisar as complicações maternas imediatas e tardias nas PB e PM; estudar os desfechos maternos e fetais de acordo os anticoagulantes utilizados na gestação e, por fim, identificar as variáveis relacionadas às complicações cardíacas e óbito materno.

## Métodos

Trata-se de um estudo clínico prospectivo de uma coorte de 128 portadoras de próteses valvares matriculadas na Unidade de Cardiopatias Valvares do Instituto do Coração-HCFMUSP que foram consecutivamente incluídas no Registro-InCor de Cardiopatia e Gravidez, entre 2017 e 2021, após a confirmação da gestação.

Setenta e oito pacientes apresentavam prótese de pericárdio bovino e formaram o grupo PB e as demais 50, tinham prótese St Jude Medical e compuseram o grupo PM. Dentre as 128 pacientes, 71 (55,4%) faziam uso de anticoagulantes antes da gestação, sendo 49 do grupo PM e 22 do grupo PB que apresentavam fibrilação atrial permanente (grupo PB+FA). Todas as pacientes tiveram aconselhamento reprodutivo prévio e foram informadas sobre os riscos da gestação e o protocolo a ser seguido na eventual ocorrência da gravidez.

**Figura 1:**
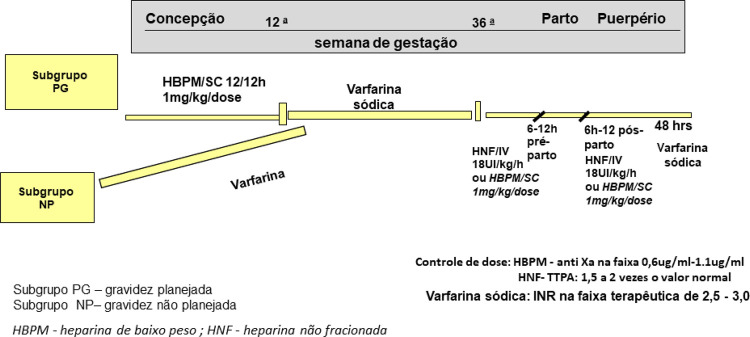
– Protocolo para o uso de Anticoagulantes Gravidez e Puerpério. Prótese mecânica e Prótese Biológica + Fibrilação atrial permanente (PB+FA).

As pacientes que planejaram a gravidez foram orientadas em adotar o protocolo sequencial de anticoagulantes, como mostra a Figura 1. Nessa orientação a heparina de baixo peso molecular (HBPM) foi empregada na dose de 1 mg/kg a cada 12hrs, e monitorada pela dosagem do fator anti Xa, semanal, com alvo terapêutico entre 0,6 e 1,1 ug/ml; enquanto a dose de varfarina foi monitorada pelo International Normalized Ratio (INR), quinzenal, com metas entre 2.5 a 3.5.

Na 1ª consulta da gestação, considerou-se as seguintes características basais: idade materna, posição anatômica da prótese, tempo decorrido entre o implante da prótese valvar e a gestação (ΔT), etiologia da doença valvar, eventos cardíacos pré-existentes à gestação (insuficiência cardíaca, acidente tromboembólico, fibrilação atrial e endocardite infecciosa), função da prótese valvar, disfunção ventricular esquerda e o tipo de anticoagulante em uso. Nessa questão, pacientes que planejaram a gravidez e adotaram o protocolo sequencial de anticoagulante formaram o subgrupo PG e as demais formaram o subgrupo NP.

O ecocardiograma transtorácico e/ou transesofágico foi realizado durante o estudo com objetivo de analisar o estado funcional da prótese (degeneração/calcificação ou trombose) de acordo com os critérios convencionais; e a presença ou não de disfunção ventricular esquerda considerada discreta (FEVE>45%), moderada (FEVE 35 a 45%) e importante (≤ FEVE 35%).

O sucesso da gravidez foi considerado quando a gestação alcançou o termo (parto ≥37 semanas), e o puerpério (< 42 dias pós-parto) ocorreram na ausência de complicações maternas e fetais. As complicações cardíacas maternas estudadas foram: insuficiência cardíaca; tromboembolismo; fibrilação atrial, endocardite infecciosa; necessidade de hospitalização para tratamento de complicações cardíacas, disfunção de prótese, necessidade de reoperação para substituição da prótese, morte materna até 42 dias após o parto e tardia (até 12 meses após o parto). Dentre as complicações obstétricas considerou-se abortamento espontâneo (gravidez interrompida ≤ 22 semanas), parto prematuro (idade gestacional ≤ 37 semanas), preeclampsia e hemorragia pós-parto. Entre as complicações do recém nascidos considerou-se o óbito neonatal, a prematuridade, malformações relacionadas à embriopatia varfarinica e a incidência de cardiopatias congênitas.

Este estudo foi aprovado pelo Comitê de Ética em Pesquisa SDC 4563/17/063 do Hospital das Clinicas da Faculdade de Medicina da Universidade de São Paulo.

### Análise estatística

Os cálculos foram realizados com auxílio do software R 4.2.0,^6^ e os gráficos foram construídos com apoio do ggplot2.^7^ A análise descritiva para variáveis quantitativas são apresentadas como médias e desvios padrões (média ± desvio padrão) ou medianas e intervalos interquartis (mediana [Q1; Q3]) quando as variáveis não apresentam distribuição normal pelo teste de Kolmogorov-Smirnov; para as variáveis qualitativas foram consideradas as frequências absolutas e relativas. Para testar associação entre as variáveis (qualitativas) foram realizados testes de Fisher ou Qui-quadrado, quando as variáveis possuem mais que duas categorias. Para a comparação das variáveis quantitativas foi utilizado teste-t, quando apresentaram distribuição normal ou Wilcoxon-Mann-Whitney (quando a distribuição da variável não foi normal, verificada pelo teste de Kolmogorov-Smirnov). Para os testes estatísticos, foi adotado um nível de significância de 5% e todos foram considerados como bicaudais.

Ajustou-se o modelo de regressão logística binária (ocorrência ou não ocorrência de pelo menos um dos eventos mencionados) com o objetivo de verificar em conjunto, as variáveis associadas à ocorrência de complicações cardíacas, disfunção da prótese, intervenção e/ou óbito durante e/ou após a gestação. Inicialmente, foram selecionadas variáveis que apresentaram no teste de associação com o desfecho o p-valor ≤ 0,15, através da análise univariada

A análise univariada foi aplicada para uma seleção inicial de variáveis que apresentaram o p-valor ≤ 0,15 no teste de associação com o desfecho. Foram também consideradas como variável agrupada a ocorrência de algum evento cardíaco e/ou disfunção da prótese anteriores à gestação. A seleção do melhor subconjunto de variáveis (com melhor ajuste) foi baseada na aplicação do método *stepwise*, utilizando o critério de Akaike.^8^

## Resultados

**Análise comparativa das características de base entre os grupos**: A média da idade foi maior do grupo PB (p = 0,021), o tempo transcorrido entre o implante da prótese valvar e a gestação foi maior (p < 0,001) no grupo PM; houve diferença na distribuição por etiologia com prevalência maior da etiologia reumática no grupo PB (75,6% versus 50,0%, p < 0,001); evento cardíaco e/ou disfunção de prótese foi mais frequente no PB (p < 0,001) (Tabela 1).

**Tabela 1:** – Características basais na 1ª consulta durante a gestação

Medida	Total (n = 128)	Prótese biológica (n = 78)	Prótese mecânica (n = 50)	p-valor
**Idade da paciente (anos)**	30,3 ± 6,2	31,3 ± 5,4	28,6 ± 7,0	0,021
**Posição anatômica da prótese**				0,329
Mitral	74/128 (57,8%)	45/78 (57,7%)	29/50 (58,0%)	
Aórtica	34/128 (26,6%)	20/78 (25,6%)	14/50 (28,0%)	
Mitral + Aórtica	14/128 (10,9%)	7/78 (9,0%)	7/50 (14,0%)	
Pulmonar	3/128 (2,3%)	3/78 (3,8%)	0/50 (0,0%)	
Tricúspide	3/128 (2,3%)	3/78 (3,8%)	0/50 (0,0%)	
Δ T Implante de Prótese-Gestação	7,3 ± 6,4	5,5 ± 4,1	10,2 ± 8,2	<0,001
**Etiologia da doença valvar**				< 0,001
Doença reumática	84/128 (65,6%)	59/78 (75,6%)	25/50 (50,0%)	
Cardiopatia congênita	39/128 (30,5%)	14/78 (17,9%)	25/50 (50,0%)	
Endocardite infecciosa	5/128 (3,9%)	5/78 (6,4%)	0/50 (0,0%)	
**Evento cardíaco anterior à gestação**				
Insuficiência cardíaca	14/128 (10,8%)	11/78 (14,1%)	3/50 (6,1%)	0,245
Acidente Tromboembólico	9/128 (7,0%)	6/78 (7,7%)	3/50 (6,0%)	> 0,999
Fibrilação atrial	35/128 (27,3%)	26/78 (33,3%)	9/50 (18,0%)	0,067
Endocardite infecciosa	15/128 (11,7%)	14/78 (17,9%)	1/50 (2,0%)	0,005
**Algum evento cardíaco anterior à gestação**	59/128 (46,1%)	45/78 (57,7%)	14/50 (28,0%)	0,001
**Função da Prótese**				< 0,001
com disfunção	21/128 (16,4%)	21/78 (26.9%)	0/50 (0,0%)	
sem disfunção	107/128 (83,6%)	57/78 (73.1%)	50/50 (100,0%)	
**Algum evento cardíaco/disfunção da prótese anterior à gestação**	66/128 (51,6%)	52/78 (66,7%)	14/50 (28,0%)	< 0,001
**Disfunção ventricular esquerda**				0,480
Não	105/128 (82,0%)	62/78 (79,5%)	43/50 (86,0%)	
Sim	23/128 (18,0%)	16/78 (20,5%)	7/50 (14,0%)	

Δ T: tempo transcorrido do implante da prótese à gestação em estudo.

**Análise dos eventos clínicos e/ou disfunção de prótese anteriores à gestação entre os grupos:** O sucesso da gravidez, foi alcançado em 64 (50,0%) pacientes dentre as quais 29 não apresentavam eventos cardíacos e/ou disfunção antes da gravidez, e não houve diferença entre os grupos (PB 56,4% versus PM 40,0% - p = 0,103). (Tabela 2)

**Tabela 2:** – Evolução materno-fetal durante a gestação e 12 meses pós-parto

Medida	Total (n = 128)	Prótese biológica (n = 78)	Prótese mecânica (n = 50)	p-valor
**Desfechos finais**				
**Sucesso da gravidez**^**(1)**^	64/128 (50,0%)	44/78 (56,4%)	20/50 (40,0%)	0,103
Complicações Cardíacas ou óbito materno	55/128 (43,0%)	41/78 (52,6%)	14/50 (28,0%)	0,007
**Complicações cardíacas/disfunção de prótese anterior e mantidas durante a/ após gestação**	46/128 (35,9%)	37/78 (47,4%)	9/50 (18,0%)	< 0,001
**Evolução da gestação até 42 dias pós-parto**				
Insuficiência cardíaca	21/128 (16,4%)	17/78 (21,8%)	4/50 (8,0%)	0,050
Tromboembolismo	3/128 (2,3%)	1/78 (1,3%)	2/50 (4,0%)	0,560
Fibrilação atrial	29/128 (22,7%)	24/78 (30,8%)	5/50 (10,0%)	0,009
Endocardite infecciosa	1/128 (0,8%)	1/78 (1,3%)	0/50 (0,0%)	> 0,999
Pelo menos um evento cardíaco (dentre os quatro acima)	42/128 (32,8%)	34/78 (43,6%)	8/50 (16,0%)	0,001
Uso de medicação cardiovascular	57/128 (44,5%)	44/78 (56,4%)	13/50 (26,0%)	< 0,001
Necessidade de hospitalização	33/128 (25,7%)	19/78 (24,4%)	14/50 (28,0%)	0,682
Óbito materno	5/128 (3,9%)	3/78 (3,8%)	2/49 (4,0%)	> 0,999
Complicações obstétricas	43/128 (33,6%)	22/78 (28,2%)	21/50 (42,0%)	0,127
**Tipo de parto**				0,191
Vaginal	31/107 (29,0%)	17/69 (24,6%)	14/38 (36,8%)	
Cesárea	76/107 (71,0%)	52/69 (75,4%)	24/38 (63,2%)	
Perdas fetais	23/128 (18,0%)	11/78 (14,1%)	12/50 (24,0%)	0,165
Peso do RN vivo (gramas)	2748 ± 528 (n = 103)	2771 ± 576 (n = 65)	2708 ± 437 (n = 38)	0,530
Idade gestacional do parto (semanas)	37,0 [36,0; 38,0] (n = 111)	37,0 [37,0; 37,0] (n = 73)	37,0 [36,0; 38,0] (n = 38)	0,860
**Complicações do RN**	29/105 (27,6%)	18/67 (26,9%)	11/38 (28,9%)	0,824
**Evolução 12 meses pós-parto/aborto (desconsiderando os 5 óbitos)**				
Insuficiência cardíaca	22/123 (17,9%)	16/75 (21,3%)	6/48 (12,5%)	0,238
Tromboembolismo	3/123 (2,4%)	0/75 (0,0%)	3/48 (6,2%)	0,057
Fibrilação atrial	16/123 (13,0%)	12/75 (16,0%)	4/48 (8,3%)	0,278
Endocardite infecciosa	1/123 (0,8%)	1/75 (1,3%)	0/48 (0,0%)	> 0,999
Pelo menos um evento cardíaco (dentre os quatro acima)	33/123 (26,8%)	24/75 (32,0%)	9/48 (18,8%)	0,144
**Função da Prótese – 12 meses pós-parto/aborto (desconsiderando os 5 óbitos)**				0,003
com disfunção	21/123 (17,1%)	19/75 (25,3%)	2/48 (4,2%)	
sem disfunção	102/123 (82,9%)	56/75 (74,7%)	46/48 (95,8%)	

(1) ausência de: complicações obstétricas, complicações do RN, óbito, eventos cardíacos ou disfunção para quem não tinha eventos anteriores à gravidez.

Houve 55 (43,0%) casos de pacientes com complicações cardíacas e/ou óbitos maternos, sendo que 46 (35,9%) foram registradas em pacientes que iniciaram a gravidez com eventos prévios e/ou disfunção da prótese, proporcionalmente maior no grupo PB (p < 0,001) (Tabela 2). Cinco óbitos maternos ocorreram em pacientes com eventos cardíacos prévios e concomitantes com complicações obstétricas e/ou fetais.

**Análise das complicações cardíacas/óbitos do grupo PB durante a gestação e 12 meses pós-parto**: Dezessete (21,8%) pacientes apresentaram insuficiência cardiaca na gestação, nove delas já apresentavam antes da gestação. Os casos de IC foram associados à fibrilação atrial permanente, à disfunção ventricular e à disfunção da prótese valvar. Houve um (1,3%) caso de embolia coronária em paciente com fibrilação atrial permanente, em uso de HBPM, na 16ª semana de gestação, que evoluiu para aborto espontâneo. Outra (1,3%) paciente apresentou endocardite infecciosa na 22ª semana de gestação, por agente do grupo HACEK (H. parainfluenza), evoluiu com choque séptico, e obteve boa resposta ao tratamento convencional, e alcance do parto cesárea com 37 semanas, sem intercorrências e recém-nascido saudável. Foram registrados três óbitos maternos associados à choque cardiogênico em pacientes com prótese biológica calcificada e estenose grave, dois durante a gestação e outro no pós-parto imediato. O seguimento ao longo de 12 meses pós-parto, houve um caso de endocardite infecciosa após 3 meses do parto por Coxiella Bunetti, que teve boa evolução clínica com tratamento convencional, contudo evoluiu com disfunção da prótese.

**Análise estrutural da prótese no grupo PB:** Vinte uma (26,9%) pacientes iniciaram a gravidez com disfunção da prótese biológica, nove delas que apresentavam estenose com calcificação, tiveram má evolução clínica, incluindo dois óbitos maternos, duas cirurgias cardíacas de emergência de troca valvar e um procedimento *de valve in valve.* Houve seis casos (6/57 – 10,5%) de “nova” disfunção da prótese ao longo de 12 meses pós-parto.

**Análise das complicações cardíacas e óbitos do grupo PM durante a gestação e 12 meses –parto:** Quatro (8,0%) pacientes apresentaram insuficiência cardíaca durante a gestação, das quais, três tinham disfunção ventricular importante e, duas apresentavam insuficiência cardíaca antes da gestação. Houve dois (4,0%) casos de tromboembolismo, ambos com HBPM, e dois óbitos maternos, um por trombose de prótese e outro por insuficiência cardíaca em paciente com disfunção ventricular. Nos 12 meses pós-parto, houve três (6,2%) casos de tromboembolismo, dois deles com trombose da prótese, um dos quais evolui para óbito.

**Análise estrutural da prótese no grupo PM:** Houve três casos de disfunção da PM causada por trombose da prótese, um caso durante a gestação que evoluiu para óbito materno e, dois outros casos, respectivamente no 48^o^ e 64º dia após o parto, requerendo cirurgia cardíaca de emergência

**Análise das complicações obstétricas e fetais nos grupos PB e PM:** A incidência de complicações obstétricas e nos recém nascidos vivos não foram diferentes entre os grupos (Tabela 2). No grupo PB foram registrados nove (11,5%) abortamentos espontâneos, quatro (5,1%) casos de pré-eclâmpsia, 19 (24,4%) partos prematuros e três (6,4%) casos de hemorragia pós-parto. Além dos abortos, ocorreram dois natimortos e duas mortes no período neonatal. Dentre os recém-nascidos vivos, três apresentaram malformações que corresponderam à 1) paralisia cerebral de grau leve (mãe submetida a troca valvar durante a gravidez); 2) comunicação interventricular e agenesia renal, e 3) comunicação ventricular. No grupo PM, houve 12 (24%) abortamentos espontâneos, seis (12%) partos prematuros e cinco (10%) casos de hemorragia materna pós-parto. Dos 12 abortos, sete foram em pacientes com HBPM e quatro em pacientes com varfarina sódica. Dos 38 recém-nascidos vivos, quatro (10,5%) apresentavam cardiopatias congênitas e outro (2,6%) apresentou hemorragia intraparenquimatosa cerebral identificada na 29^a^ semana de gestação, na vigência de varfarina sódica.

**Análise das complicações materna e fetais de acordo com o uso de anticoagulantes:** Setenta e uma (55,4%) pacientes utilizaram anticoagulantes, 45 (63,4%) pacientes do subgrupo PG em esquema sequencial e 26 (36,6%) pacientes do subgrupo NP (Tabela 4). Houve menor frequência de perdas fetais (8,9% versus 46,2% - p < 0,001) no subgrupo PG (Tabela 4A). Vinte e duas pacientes do grupo PB+FA tiveram menor percentual, embora não significativo, de sucesso da gravidez comparado ao grupo PB (Tabela 4B).

**Tabela 4:** – Proporção de pacientes e adesão aos esquemas de antiogulantes

	Total (n = 128)	Prótese biológica (n = 78)	Prótese mecânica (n = 50)	p-valor
**Anticoagulante - sim**	71/128 (55,4%)	22/78 (28,2%)	49/50 (98,0%)	< 0,001
**Esquema utilizado**				0,003
Protocolo sequencial (subgrupo PG)	45/71 (63,4%)	12/22 (54,5%)	33/49 (67,3%)	
HBPM durante a gestação (subgrupo NP)	11/71 (15,5%)	8/22 (36,4%)	3/49 (6,1%)	
Varfarina durante a gestação (subgrupo NP)	15/71 (21,1%)	2/22 (9,1%)	13/49 (26,5%)	

**Figura 2:**
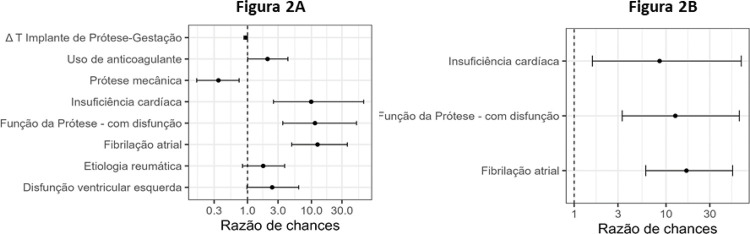
– Regressão logístico da análise univariada (Fig 2A) e multivariada (Fig 2B) para complicações cardíacas e óbito materno, considerando as variáveis anteriores à gestação.

A análise univariada selecionou o tipo da prótese (biológica ou mecânica) (odds ratio 0,35; CI 95% [0,16; 0,74]; p = 0,007), Δ T implante da prótese (odds ratio 0,94; CI 95% [0,88; 0,99]; p = 0,045), etiologia da doença valvar (reumática ou outra) (odds ratio 1,8; CI 95% [0,8; 3,8]; p = 0,144), pré-existência à gestação de insuficiência cardíaca (odds ratio 9,9; CI 95% [2,6; 65,6]; p = 0,004), fibrilação atrial (odds ratio 12,5; CI 95% [4,9; 36,5]; p < 0,001), disfunção da prótese (odds ratio 11,4; CI 95% [3,6; 50,7]; p < 0,001), disfunção ventricular esquerda (odds ratio 2,4; CI 95% [0,98; 6,3]; p = 0,06), e o uso de anticoagulantes (odds ratio 2,1; CI 95% [1,0; 4,3]; p = 0,05), como variáveis preditivas de complicações cardíacas e óbito materno (Tabela 3 - Figura 2A).

**Tabela 3:** – Complicações cardíacas e óbito materno de acordo com as características de base antes da gestação

Medida	Total (n = 128)	Complicação ou óbito materno		p-valor
		Não (n = 73)	Sim (n = 55)	
**Prótese**				0,007
Biológica	78/128 (60,9%)	37/73 (50,7%)	41/55 (74,5%)	
Mecânica	50/128 (39,1%)	36/73 (49,3%)	14/55 (25,5%)	
**Idade da paciente (anos)**	30,3 ± 6,2	29,9 ± 6,7	30,8 ± 5,4	0,156
**Posição anatômica da prótese**				0,157
Mitral	74/128 (57,8%)	37/73 (50,7%)	37/55 (67,3%)	
Aórtica	34/128 (26,6%)	21/73 (28,8%)	13/55 (23,6%)	
Mitral + Aórtica	14/128 (10,9%)	9/73 (12,3%)	5/55 (9,1%)	
Pulmonar	3/128 (2,3%)	3/73 (4,1%)	0/55 (0,0%)	
Tricúspide	3/128 (2,3%)	3/73 (4,1%)	0/55 (0,0%)	
Δ T Implante de Prótese-Gestação	7,3 ± 6,4	8,3 ± 6,7	6,0 ± 5,8	0,051
**Etiologia da doença valvar**				0,089
Doença reumática	84/128 (65,6%)	44/73 (60,3%)	40/55 (72,7%)	
Cardiopatia congênita	39/128 (30,5%)	24/73 (32,9%)	15/55 (27,3%)	
Endocardite infecciosa	5/128 (3,9%)	5/73 (6,8%)	0/55 (0,0%)	
**Evento cardíaco anterior à gestação**				
Insuficiência cardíaca	14/128 (10,9%)	2/73 (2,7%)	12/55 (21,8%)	< 0,001
Acidente Tromboembólico	9/128 (7,0%)	3/73 (4,1%)	6/55 (10,9%)	0,171
Fibrilação atrial	35/128 (27,3%)	6/73 (8,2%)	29/55 (52,7%)	< 0,001
Endocardite infecciosa	15/128 (11,7%)	10/73 (13,7%)	5/55 (9,1%)	0,581
**Função da Prótese**				< 0,001
com disfunção	21/128 (16,4%)	3/73 (4,1%)	18/55 (32,7%)	
sem disfunção	107/128 (83,6%)	70/73 (95,9%)	37/55 (67,3%)	
**Algum evento cardíaco/disfunção da prótese anterior à gestação**	66/128 (51,6%)	20/73 (27.4%)	46/55 (83.6%)	< 0,001
**Disfunção ventricular esquerda**				0,066
Não	105/128 (82,0%)	64/73 (87,7%)	41/55 (74,5%)	
Sim	23/128 (18,0%)	9/73 (12,3%)	14/55 (25,5%)	
**Uso de anticoagulante**				0,072
Não	57/128 (44,5%)	38/73 (52,1%)	19/55 (34,5%)	
Sim	71/128 (55,5%)	35/73 (47,9%)	36/55 (65,5%)	

Δ T: tempo transcorrido do implante da prótese à gestação em estudo.

A análise multivariada mostrou que no modelo conjunto, a pré-existência de insuficiência cardíaca (odds ratio 8,5; CI 95% [1,4; 50,7]; p = 0,019), fibrilação atrial (odds ratio 16,7; CI 95% [5,7; 49,1]; p < 0,001) e disfunção da prótese biológica (odds ratio 12,6; CI 95% [3,0; 52,7]; p = 0,001) foram as variáveis de maior força de predição de complicações e/ou óbitos maternos (Tabela 5 - Figura 2B).

**Tabela 5:** – Resultado do modelo final para atividade remunerada

Coeficiente	Estimativa	Erro padrão	Razão de chances (RC)	IC 95%		p-valor
Intercepto	-1,6	0,3				< 0,001
Insuficiência cardíaca - sim	2,1	0,9	8,5	1,4	50,7	0,019
Fibrilação atrial - sim	2,8	0,6	16,7	5,7	49,1	< 0,001
Função da Prótese - com disfunção	2,5	0,7	12,6	3,0	52,7	0,001

## Discussão

O presente estudo, que analisou 128 gestantes portadoras de próteses valvares, mostrou que a taxa de gestações a termo, sem complicações cardíacas, obstétricas e/ou fetais, não ultrapassou a metade dos casos estudados.

Esses resultados reforçam o conceito da literatura mundial de que mulheres com próteses valvares, sejam elas quais forem, têm baixa taxa de sucesso na gestação,^2,3,5,9^ o que pode ser atribuído a fatores, tais como, complicações naturais da doença valvar, disfunção estrutural da prótese e riscos relacionados ao uso de anticoagulantes.

O destaque desta coorte, foi a proporção de 65% de casos de etiologia reumática, indicativo de uma realidade persistente no Brasil, aonde a ocorrência de doença valvar em mulheres jovens tem forte associação com a doença reumática.

É oportuno lembrar, que a sobrecarga cardiocirculatória e o estado de hipercoagulabilidade, fisiológicos da gestação e do puerpério,^10,11^ são os determinantes de impacto na ocorrência de complicações em mulheres portadoras de próteses valvares.

Neste estudo, a padronização da prótese biológica de pericárdio bovino em 78 mulheres e a mecânica de duplo folheto (St.Jude Medical) nas demais 50 pacientes, seguiu uma prática protocolar da Instituição, considerando as condições clínicas no momento da cirurgia, os melhores resultados em termos de sobrevida livre de disfunção da prótese e a decisão compartilhada com a paciente.^12-14^

**Evolução clínica e estrutural da prótese biológica (Grupo PB):** Foi notável que a prevalência de 75,6% de reumáticas no grupo PB, certamente justificou a alta frequência e a combinação de eventos cardíacos pré-existentes à gestação, tais como fibrilação atrial e insuficiência cardíaca, sequelas atribuíveis à evolução lenta e insidiosa da pancardite reumatismal, ao longo de décadas.^15^

Entre as características do grupo PB apresentadas na Tabela 1, nota-se que as pacientes iniciaram a gravidez com um “estigma” clinico desfavorável para a evolução da gestação, o que acarretou um número maior de complicações maternas nesse grupo (p = 0,007 - Tabela 3). De fato, o presente estudo também demonstrou que eventos cardíacos pré-existentes foram fatores de predição das complicações cardíacas e óbito materno, durante a gestação e após o parto. (Tabela 5 - Figura 2)

É destaque na literatura mundial, o pior prognóstico materno e fetal de gestantes portadoras de cardiopatias que vivem em países emergentes com baixo e médio nível socioeconômico e cultural da população.^16,17^ Nesse cenário, o Registro Indiano de Cardiopatia e Gravidez (M-Pac) mostrou uma prevalência de 42,1% de doença valvar reumática entre 1029 gestantes portadoras de cardiopatias, e documentou um percentual de morte materna (8,6%) e de complicações cardíacas (34,3%) significativamente maior entre as portadoras de próteses valvares quando comparado a outras lesões cardíacas estruturais.^18^

No entanto, o estudo holandês de Lameijer et al.^19^ que incluiu 78 gestantes com próteses valvares somente de etiologia congênita, mostrou 17% de complicações cardíacas com predomínio nas próteses mecânicas, e chamou a atenção para a disfunção de prótese biológicas pré-existente à gestação como marcador de alto risco para as complicações na gravidez.

Do ponto de vista estrutural, o presente estudo verificou um pior desempenho das próteses biológicas, dado que no tempo médio de cinco anos após o implante, foi documentado 27% de disfunções estruturais das próteses identificadas na primeira consulta da gestação, e que tiveram alta significância no valor preditivo de má evolução materna (Figura 2).

Esses resultados, foram concordantes com Wichert-Schmitt et al.,^20^ que registraram 27% de disfunções de próteses biológicas no início da gestação de uma coorte de 125 pacientes, com significativa correlação entre a disfunção e a pior evolução materno-fetal, particularmente com as próteses localizadas à esquerda do coração.

Ademais, o ponto crucial do presente estudo, foram as graves consequências das disfunções calcificadas nas próteses biológicas, o que resultou em três óbitos maternos consequentes ao choque cardiogênico. Diante desses resultados, a atual opinião dos especialistas, é favorável à cirurgia de reoperação para substituição da prótese biológica, quando calcificada, seja durante a gestação, mas idealmente no planejamento de “nova” gravidez, mesmo em pacientes assintomáticas.^21^

Este estudo também trouxe à tona uma discussão controversa ao evidenciar cinco casos de disfunção “nova” da prótese ao longo de 12 meses pós-parto, uma delas causada pela endocardite infecciosa, três meses após o parto. Este resultado deve ser atribuído a sobrevida natural da prótese biológica ao longo dos anos, uma vez que já ficou demonstrado que gravidez não exerce influência na durabilidade das próteses biológicas.^22-24^

**Evolução clínica e estrutural da prótese mecânica (Grupo PM):** Como contrapartida, as pacientes do grupo PM eram mais jovens, 50% delas tinham etiologia congênita, e a despeito do maior tempo decorrido desde o implante da prótese, essas pacientes iniciaram a gravidez com menos fatores complicadores, o que a princípio indicava um curso clinico mais favorável (Tabela 3).

No entanto, a despeito da ausência de disfunção da prótese mecânica prévia e das características clinicas mais favoráveis no início da gestação, somente 40% alcançaram o sucesso da gravidez, lembrando as três ocorrências de trombose de prótese, uma no primeiro trimestre da gestação com desfecho fatal, e duas após o parto, exigindo intervenção cirúrgica de emergência. A complexidade desta situação reforça a importância da supervisão que não se limita à gestação, mas deve-se estender ao período pós-parto.

Estima-se que 5 a 20% das pacientes apresentam complicações tromboembólicas e/ou hemorrágicas durante gestação, parto ou puerpério, por conta do uso dos anticoagulantes.^25-27^ O Registro Europeu de Cardiopatia e Gravidez da Sociedade Europeia de Cardiologia-ROPAC, que incluiu 212 gestantes com próteses mecânicas, mostrou que apenas 58% delas tiveram gravidez sem complicações, sendo que a trombose da prótese ocorreu em 4,7% (IC 95%, 3,06-7,26) dos casos com uma taxa de 18% de morte materna.^28^

À vista disso, o Posicionamento de Cardiopatia e Gravidez da Sociedade Brasileira de Cardiologia^21^ e a Organização Mundial de Saúde classifica pacientes com próteses mecânicas em alto risco para a gravidez (Classe III-OMSm)^29^e recomenda que essas gestações devam ser acompanhadas em hospital terciário com equipe multidisciplinar especializada.

**Protocolo para o uso dos anticoagulantes**: A orientação sobre o uso de anticoagulantes na gravidez envolve a ponderação entre os riscos da trombose e hemorragia materna, e os riscos teratogênicos e hemorrágicos do feto. Atualmente, não há um posicionamento sobre o melhor esquema de anticoagulantes para a gestantes portadoras de cardiopatias, visto que nenhuma opção farmacológica, isolada ou combinada, fornece evidências sobre a eficácia em não acarretar efeitos adversos à mãe e/ou ao feto

O protocolo adotado neste estudo, foi apoiado na literatura científica, validada pelos melhores resultados e embasada na experiência da Instituição.^30,31^ Do ponto de vista da bioética, houve a preocupação constante em manter o benefício materno dos anticoagulantes, e evitar o maleficio dos seus efeitos teratogênicos (Figura 1).

Nesse sentido, os estudos clínicos defendem a eficácia da varfarina sódica na prevenção de tromboembolismo nas próteses mecânicas ou doença valvar com fibrilação atrial e, ao mesmo tempo, desencorajam o uso da HBPM dada sua associação com a alta incidência de eventos tromboembólicos e morte materna.^25-27^

De fato, o presente estudo registrou 6,5% de casos de trombose de prótese com uso de HBPM, a despeito do ajuste das doses de acordo com os níveis plasmáticos entre 1,0 e 1,2 UI/ml da atividade do fator anti-Xa. Na prática, há sérias dificuldades para o alcance das metas terapêuticas com HBPM, vez que os níveis do fator anti Xa sofrem importantes flutuações nas 24 horas.^32^

O estudo prospectivo que incluiu 15 gestantes em uso de doses terapêutica de enoxaparina, mostrou que os níveis médios de atividade anti fator Xa foram subterapêuticos em 73% (11/15) dos casos, condição que pode explicar os eventos trombóticos associados a HBPM.^33^

Vale comentar que a orientação que as pacientes receberam no pós-operatório do implante da prótese, quanto aos riscos das eventuais complicações e o esquema recomendado perante a ocorrência de gravidez, foi a diferencial neste estudo. O aconselhamento antes da concepção adequado, incluindo a orientação à contracepção, não é a realidade, estima-se que apenas 5% das mulheres com próteses valvares cardíacas são orientadas para o uso de métodos contraceptivos e que cerca de 5% a 10% das mulheres tem a conscientização do diagnóstico da sua cardiopatia.^1,16,28^

Mesmo com a orientação previa obtida em nosso estudo, 26 (36,6%) das pacientes não utilizaram o esquema sequencial recomendado, seja pelo acontecimento da gravidez não programada ou por decisões pessoais, e acabaram sofrendo um número maior de abortos espontâneos (Tabela 4A). Curiosamente as complicações cardíacas e óbitos maternos não foram maiores entre as pacientes que usaram anticoagulantes (Tabela 3). No entanto as perdas fetais e as complicações cardíacas entre essas pacientes foram maiores no subgrupo NP e grupo PB+FA, respectivamente (Tabela 4A e 4B).

**Tabela 4:** A – Análise das complicações materno fetais nos subgrupos PG vs. NP

	Total em com anticoagulação (n = 71)	Esquema anticoagulação		p-valor
		PG (n = 45)	NP (n = 26)	
**Prótese**				0,425
Biológica	22/71 (31,0%)	12/45 (26,7%)	10/26 (38,5%)	
Mecânica	49/71 (69,0%)	33/45 (73,3%)	16/26 (61,5%)	
**Complicações maternas (incluindo óbito)**	36/71 (50,7%)	21/45 (46,7%)	15/26 (57,7%)	0,462
**Perdas fetais**	16/71 (22,5%)	4/45 (8,9%)	12/26 (46,2%)	< 0,001
**Complicações do recém-nascido**	17/55 (30,9%)	12/41 (29,3%)	5/14 (35,7%)	0,742
**Sucesso na gravidez**	30/71 (42,3%)	22/45 (48,9%)	8/26 (30,8%)	0,212

**Tabela 4:** B – Análise das complicações materno-fetais no grupo PB versus PB+FA

	Total PB (n = 78)	Em uso de anticoagulante		p-valor
		Não (n = 56)	Sim (n = 22)	
Complicações maternas (incluindo óbito)	41/78 (52,6%)	19/56 (33,9%)	22/22 (100,0%)	< 0,001
Perdas fetais	11/78 (14,1%)	6/56 (10,7%)	5/22 (22,7%)	0,276
Complicações do recém-nascido	18/67 (26,9%)	12/50 (24,0%)	6/17 (35,3%)	0,364
Sucesso na gravidez	44/78 (56,4%)	34/56 (60,7%)	10/22 (45,5%)	0,311

Protocolo sequencial – HBPM no primeiro trimestre, varfarina sódica, do segundo trimestre à 36ª semana de gestação; Grupo PG – planejou a gravidez; Grupo NP – não planejou gravidez; HBPM – heparina de baixo peso molecular, grupo PB – prótese biológica sem uso de anticoagulante grupo PB+FA prótese biológica associada a fibrilação atrial em uso de anticoagulante.

**Complicações obstétricas e fetais**: É provável que alta incidência de complicações obstétricas e fetais, especialmente no grupo que não seguiu o esquema sequencial (Tabela 4B), seja a consequência do uso da varfarina sódica, que pode causar abortos espontâneos em cerca de 10% a 30% dos casos.^34^ Além disso, a varfarina sódica é mencionada como teratogênica e causa malformações conhecidas como síndrome do “varfarina fetal,” independente da dose, não obstante, não tenha sido identificada em nenhum caso neste estudo.^35-37^

Outra complicação, é hemorragia intracraniana fetal, pouco frequente, geralmente acontece no segundo trimestre da gestação e nas faixas terapêuticas da anticoagulação com a varfarina sódica.^38^ Embora seja fatal na maioria dos casos, esses acidentes hemorrágicos, podem causar sequelas graves nos recém-nascidos sobreviventes, como ocorreu no presente estudo. Vale lembrar que a farmacocinética da varfarina sódica resulta em uma alta concentração sanguínea no feto, que não pode ser estimada com precisão em exames do sangue materno.^39^

Outro dado que chamou a atenção foi a identificação de 5,8% de cardiopatias congênitas nos recém-nascido vivos, taxa cinco vezes superior à prevalência de 0,8% a 1,0% na população geral.^40^ Talvez, o caráter hereditário das cardiopatias congênitas presente nesta casuística tenha contribuído para essa alta incidência, além do uso da varfarina sódica que não pode ser descartada.

Ainda merece destaque a ocorrência de um caso de comprometimento neurológico do feto decorrente da cirurgia cardíaca durante o segundo trimestre da gestação. Esta grave complicação, já descrita na literatura, tem como a hipótese a sequela neurológica causada pela anoxia cerebral transitória intraútero em resposta ao fluxo não pulsátil e laminar, à hemodiluição e à hipotermia utilizadas na circulação extracorpórea.^41^

## Considerações finais

O número de participantes deste estudo não foi grande, e, por ser conduzido em um único centro de referência em cardiologia, não é isento de influências de seleção nas pacientes. Contudo, é possível destacar diferenças na metodologia que realçam a qualidade e a abrangência desta pesquisa, tais como: 1) acompanhamento das pacientes desde o implante da prótese valvar; 2) o aconselhamento reprodutivo antes da gestação; 3) orientação sobre o esquema de anticoagulantes a ser seguido no início da gestação; 4) a padronização das próteses na consistência dos resultados; 5) seguimento das pacientes até 12 meses após o parto, período considerado morte materna tardia, e 6) período de estudo foi limitado a cinco anos para evitar mudanças na prática clínica ao longo do tempo.

## Conclusões

Portadoras de próteses valvares tem baixo índice de sucesso na gravidez. Os fatores complicadores da doença valvar, a sobrevida estrutural limitada das próteses biológicas e a inexistência de anticoagulantes que assegurem a evolução da gestação, são os grandes obstáculos ao êxito materno/fetal. A escolha da prótese na intenção de uma futura gravidez, não pode ser vista como uma decisão isolada, mas sim fazer parte integrante de cardiopatias que apresentam evolução complexa. O baixo índice de sucesso demonstrado neste estudo, merece reflexões no planejamento de uma gestação e motiva a continuidade das pesquisas em mulheres portadoras de doença valvar em idade reprodutiva.

## References

[1] Millar LM, Lloyd G, Bhattacharyya S (2022). Care of the Patient after Valve Intervention. Heart.

[2] Grashuis P, Khargi SDM, Veen K, El Osrouti A, Bemelmans-Lalezari S, Cornette JMJ (2023). Pregnancy Outcomes in Women with a Mitral Valve Prosthesis: A Systematic Review and Meta-Analysis. JTCVS Open.

[3] Ranjan R, Adhikary D, Adhikary AB (2019). Pregnancy Outcome with Prosthetic Heart Valve in Bangladesh: A Retrospective Cohort Study. Mymensingh Med J.

[4] Lawley CM, Lain SJ, Algert CS, Ford JB, Figtree GA, Roberts CL (2015). Prosthetic Heart Valves in Pregnancy, Outcomes for Women and Their Babies: A Systematic Review and Meta-Analysis. BJOG.

[5] Batra J, Itagaki S, Egorova NN, Chikwe J (2018). Outcomes and Long-Term Effects of Pregnancy in Women with Biologic and Mechanical Valve Prostheses. Am J Cardiol.

[6] R Core Team (2024). R: A language and environment for statistical computing.

[7] Wickham H (2009). Ggplot2: Elegant Graphics for Data Analysis.

[8] Montgomery DC, Peck EA, Vining GG (2001). Introduction to Linear Regression Analysis.

[9] Makhija N, Tayade S, Tilva H, Chadha A, Thatere U (2022). Pregnancy after Cardiac Surgery. Cureus.

[10] Soma-Pillay P, Nelson-Piercy C, Tolppanen H, Mebazaa A (2016). Physiological Changes in Pregnancy. Cardiovasc J Afr.

[11] Kohlhepp LM, Hollerich G, Vo L, Hofmann-Kiefer K, Rehm M, Louwen F (2018). Physiological Changes During Pregnancy. Anaesthesist.

[12] Fernandes JRC, Sampaio RO (2021). Mechanical Prosthesis X Biological Prosthesis: an Individualized and Shared Decision. Arq Bras Cardiol.

[13] Brandão CMA, Pomerantzeff PMA, Cunha CR, Morales JIE, Puig LB, Grinberg M (2000). Substituição Valvar com Próteses Mecânicas de Duplo Folheto. Rev Bras Cir Cardiovasc.

[14] Tarasoutchi F, Montera MW, Ramos AIO, Sampaio RO, Rosa VEE, Accorsi TAD (2017). Atualização das Diretrizes Brasileiras de Valvopatias: Abordagem das Lesões Anatomicamente Importantes. Arq Bras Cardiol.

[15] Moorthy PSK, Sivalingam S, Dillon J, Kong PK, Yakub MA (2019). Is it Worth Repairing Rheumatic Mitral Valve Disease in Children? Long-Term Outcomes of an Aggressive Approach to Rheumatic Mitral Valve Repair Compared to Replacement in Young Patients. Interact Cardiovasc Thorac Surg.

[16] Beaton A, Okello E, Scheel A, DeWyer A, Ssembatya R, Baaka O (2019). Impact of Heart Disease on Maternal, Fetal and Neonatal Outcomes in a Low-Resource Setting. Heart.

[17] Roos-Hesselink J, Baris L, Johnson M, De Backer J, Otto C, Marelli A (2019). Pregnancy Outcomes in Women with Cardiovascular Disease: Evolving Trends Over 10 Years in the ESC Registry Of Pregnancy And Cardiac Disease (ROPAC). Eur Heart J.

[18] Paul GJ, Princy SA, Anju S, Anita S, Mary MC, Gnanavelu G (2023). Pregnancy Outcomes in Women with Heart Disease: The Madras Medical College Pregnancy And Cardiac (M-PAC) Registry from India. Eur Heart J.

[19] Lameijer H, van Slooten YJ, Jongbloed MRM, Oudijk MA, Kampman MAM, van Dijk AP (2018). Biological versus Mechanical Heart Valve Prosthesis During Pregnancy in Women with Congenital Heart Disease. Int J Cardiol.

[20] Wichert-Schmitt B, Grewal J, Malinowski AK, Pfaller B, Losenno KL, Kiess MC (2022). Outcomes of Pregnancy in Women with Bioprosthetic Heart Valves with or Without Valve Dysfunction. J Am Coll Cardiol.

[21] Avila WS, Alexandre ERG, Castro ML, Lucena AJG, Marques-Santos C, Freire CMV (2020). Brazilian Cardiology Society Statement for Management of Pregnancy and Family Planning in Women with Heart Disease - 2020. Arq Bras Cardiol.

[22] Avila WS, Rossi EG, Grinberg M, Ramires JA (2002). Influence of Pregnancy after Bioprosthetic Valve Replacement in Young Women: A Prospective Five-Year Study. J Heart Valve Dis.

[23] Cleuziou J, Hörer J, Kaemmerer H, Teodorowicz A, Kasnar-Samprec J, Schreiber C (2010). Pregnancy does Not Accelerate Biological Valve Degeneration. Int J Cardiol.

[24] El SF, Hassan W, Latroche B, Helaly S, Hegazy H, Shahid M (2005). Pregnancy Has no Effect on the Rate of Structural Deterioration of Bioprosthetic Valves: Long-Term 18-Year Follow up Results. J Heart Valve Dis.

[25] Chan WS, Anand S, Ginsberg JS (2000). Anticoagulation of Pregnant Women with Mechanical Heart Valves: A Systematic Review of the Literature. Arch Intern Med.

[26] D'Souza R, Ostro J, Shah PS, Silversides CK, Malinowski A, Murphy KE (2017). Anticoagulation for Pregnant Women with Mechanical Heart Valves: A Systematic Review and Meta-Analysis. Eur Heart J.

[27] Romera AE, Sánchez JJ, Sierra JN, Paracuellos TS, Salguero FJV, Solanilla BR (2021). Mitral Valve Thrombosis in Term Pregnancy: A Case Report and Review of the Literature. Taiwan J Obstet Gynecol.

[28] van Hagen IM, Roos-Hesselink JW, Ruys TP, Merz WM, Goland S, Gabriel H (2015). Pregnancy in Women with a Mechanical Heart Valve: Data of the European Society of Cardiology Registry of Pregnancy and Cardiac Disease (ROPAC). Circulation.

[29] van Hagen IM, Boersma E, Johnson MR, Thorne SA, Parsonage WA, Subías PE (2016). Global Cardiac Risk Assessment in the Registry Of Pregnancy And Cardiac Disease: Results of a Registry from the European Society of Cardiology. Eur J Heart Fail.

[30] Avila WS, Grinberg M (2005). Anticoagulation, Pregnancy and Cardiopathy. A triad, Three Dominions and Five Moments. Arq Bras Cardiol.

[31] Ali M, Becker RC (2020). Bridging Anticoagulation with Mechanical Heart Valves: Current Guidelines and Clinical Decisions. Curr Cardiol Rep.

[32] van den Broek MPH, Verschueren MV, Knibbe CAJ (2022). Critical Appraisal of Evidence for Anti-Xa Monitoring and Dosing of Low-Molecular-Weight Heparin in Renal Insufficiency. Expert Rev Clin Pharmacol.

[33] Friedrich E, Hameed AB (2010). Fluctuations in Anti-Factor Xa Levels with Therapeutic Enoxaparin Anticoagulation in Pregnancy. J Perinatol.

[34] van Driel D, Wesseling J, Sauer PJ, Touwen BC, van der Veer E, Heymans HS (2002). Teratogen Update: Fetal Effects after in Utero Exposure to Coumarins Overview of Cases, Follow-Up Findings, and Pathogenesis. Teratology.

[35] Basu S, Aggarwal P, Kakani N, Kumar A (2016). Low-Dose Maternal Warfarin Intake Resulting in Fetal Warfarin Syndrome: In Search for a Safe Anticoagulant Regimen During Pregnancy. Birth Defects Res A Clin Mol Teratol.

[36] Bian C, Wei Q, Liu X (2012). Influence of Heart-Valve Replacement of Warfarin Anticoagulant Therapy on Perinatal Outcomes. Arch Gynecol Obstet.

[37] Hassouna A, Allam H (2014). Limited Dose Warfarin Throughout Pregnancy in Patients with Mechanical Heart Valve Prosthesis: A Meta-Analysis. Interact Cardiovasc Thorac Surg.

[38] Matsuda Y, Hashiguchi K, Akizawa Y, Saito R, Ohta H (2003). A Case of Fetal Subdural Hematoma at 31 Weeks of Gestation in a Woman on Warfarin Therapy after Cabrol's Operation. Fetal Diagn Ther.

[39] Reverdiau-Moalic P, Delahousse B, Body G, Bardos P, Leroy J, Gruel Y (1996). Evolution of Blood Coagulation Activators and Inhibitors in the Healthy Human Fetus. Blood.

[40] Soares AM (2020). Mortality in Congenital Heart Disease in Brazil - What do we Know?. Arq Bras Cardiol.

[41] Avila WS, Gouveia AM, Pomerantzeff P, Bortolotto MR, Grinberg M, Stolf N (2009). Maternal-Fetal Outcome and Prognosis of Cardiac Surgery During Pregnancy. Arq Bras Cardiol.

