# Supplementary material for: A Escolha da Prótese Valvar para o Sucesso da Gravidez. A “Ponta do Iceberg” para uma Doença de Evolução Complexa
Source: Arq Bras Cardiol. 2025 Feb 20;122(1):e20240163. [Article in Portuguese] doi: 10.36660/abc.20240163 (PMC11870119; doi:10.36660/abc.20240163)
Supplement: Translation

# Translation: Choosing a Valve Prosthesis for a Successful Pregnancy. The “Tip of the Iceberg” for a Disease of Complex Evolution

**DOI:** 10.36660/abc.20240163i

**Authors:** Walkiria Samuel Avila, Daniel Vinicius Rodrigues Pinto, Jessica Sol Brugnara, Marilia Moro, Talita Carla Stratti Moreira, Isabelle Borges, Nana Miura, Flávio Tarasoutchi

**Affiliations:** 1 Brazil Instituto do Coração do Hospital das Clínicas da Faculdade de Medicina da Universidade de São Paulo, São Paulo, SP – Brazil

**Keywords:** Heart Valve Prosthesis, Pregnancy, Maternal Death, Anticoagulants

## Abstract

**Background:**

The choice of valve prosthesis in women planning a pregnancy is still controversial. The durability of biological prostheses and the characteristic thrombogenic of mechanical prostheses are limitations to the pregnancy’s successful Objectives: To study the pregnancy success rate after valve prosthesis implantation, and identify the variables related to maternal outcomes.

**Methods:**

Prospective study with 78 pregnant women with bovine pericardial prosthesis (Group BP) and 50 with a mechanical prosthesis (Group MP), who received prior guidance on the risks of pregnancy. The pregnancy success rate was considered in the absence of complications cardiac, obstetric and/or fetal complications.

**Results:**

Successful pregnancy was achieved in 64 (50.0%) patients, not differing between groups (BP 56.4% vs MP 40.0% - p=0.103). The BP group had a higher cardiac events rate and prosthesis dysfunction (43.6% vs 16.0% p<0.001; 26.9% vs 2.0% p<0.001). The frequency of fetal losses (14.1% vs 24.0% p=0.165) and obstetric complications (28.2% vs 42% p=0.127) were not different between the BP and MP groups. The pre-existence of heart failure (odds ratio 8.5; 95% CI [1.4; 50.7]; p=0.019), atrial fibrillation (odds ratio 16.7; 95% CI [5.7; 49 .1]; p<0.001) and dysfunction of the biological prosthesis (odds ratio 12.6; 95% CI [3.0; 52.7]; p=0.001) were the variables predicting complications and/or deaths

**Conclusions:**

Patients with valve prostheses had low maternal-fetal success due to the complicating factors of valve disease, the limited structural survival of biological prostheses and the lack of anticoagulants to guarantee pregnancy. The choice of a prosthesis, whether biological or mechanical, should not be considered an isolated decision, but rather a consequence of a complex outcome of the heart disease.

**Central Illustration:** Choosing a Valve Prosthesis for a Successful Pregnancy. The “Tip of the Iceberg” for a Disease of Complex Evolution

## Introduction

The implantation of prosthetic valves has made it possible to carry out pregnancies in patients with severe structural heart lesions as a result of rheumatic and congenital valve disease.

The choice of valve replacement for women of reproductive age must take into account that both prostheses, biological (BP) and mechanical (MP), have particularities that determine maternal and fetal risks, in view of a future pregnancy.

The limited durability of BP in young women and the need for reoperation are the major obstacles to their choice in the reproductive age group. On the other hand, the thrombogenic nature of the MP and the hypercoagulable state of the pregnancy and puerperium require the permanent and effective use of anticoagulants, despite the serious adverse effects on pregnancy.

The limitations inherent in most studies on valve prostheses and pregnancy are related to their retrospective character, heterogeneous casuistry and limitations in the methodology, which prevent optimal accuracy in validating conclusions.^1-5^

These inaccuracies motivated this study on pregnancies in women after valve prosthesis implantation, which followed strict criteria for standardizing the valve substitute, the care conduct prior to pregnancy and up to 12 months after delivery, and the protocol for the use of anticoagulants.

The main objective of the study was to evaluate women during pregnancy and up to 12 months after delivery, after valve prosthesis implantation, and to compare the evolution between BP and MP. The secondary objectives were: to analyze immediate and late maternal complications in BP and MP; to study maternal and fetal outcomes according to the anticoagulants used during pregnancy and, finally, to identify the variables related to cardiac complications and maternal death.

## Methods

This is a prospective clinical study of a cohort of 128 valve prosthesis patients enrolled in the Valve Heart Disease Unit of Heart Institute-HCFMUSP who were consecutively included in the Heart Disease and Pregnancy: Registry InCor, between 2017 and 2021, after confirmation of pregnancy.

Seventy-eight patients had a bovine pericardial prosthesis and formed the BP group and the other 50 had a St Jude Medical prosthesis and made up the MP group. Of the 128 patients, 71 (55.4%) were using anticoagulants before pregnancy, 49 in the MP group and 22 in the BP group who had permanent atrial fibrillation (BP+AF group). All the patients had received prior reproductive counseling and were informed about the risks of pregnancy and the protocol to be followed in the event of pregnancy.

Patients planning pregnancy were instructed to adopt a sequential anticoagulant protocol, as shown in Figure 1. In this guideline, low molecular weight heparin (LMWH) was used at a dose of 1mg/kg every 12hrs, and monitored by the dosage of anti-factor Xa, weekly, with a therapeutic target between 0.6 and 1.1 IU/ml; while the dose of warfarin was monitored by the International Normalized Ratio (INR), every two weeks, with targets between 2.5 and 3.5.

**Figure 1:** – Protocol for aanticoagulantion during pregnancy and puerperium. Mechanical prosthesis and Biological prosthesis + Atrial Fibrilattion (BP + AF).

At the first appointment during pregnancy, the following baseline characteristics were considered: maternal age, anatomical position of the prosthesis, time elapsed between implantation of the valve prosthesis and pregnancy (ΔT), etiology of the valve disease, cardiac events prior to pregnancy as heart failure (HF), thromboembolic accident, atrial fibrillation (AF) and infective endocarditis, function of the valve prosthesis, left ventricular dysfunction and the type of anticoagulant being used. In this question, patients who planned pregnancy and adopted the sequential anticoagulant protocol formed the PG subgroup and the others formed the NP subgroup.

Transthoracic and/or transesophageal echocardiography was performed during the study in order to analyze the functional status of the prosthesis (degeneration/calcification or thrombosis) according to conventional criteria; and the presence or absence of left ventricular dysfunction considered mild (LVEF>45%) , moderate ( LVEF 35 to 45%) and important (≤LVEF 35%).

Pregnancy success was considered when pregnancy reached term (delivery ≥37 weeks), and the puerperium (<42 days postpartum) occurred in the absence of maternal and fetal complications. The maternal cardiac complications studied were: HF; thromboembolism; AF, infective endocarditis; need for hospitalization to treatement of cardiac complications, prosthesis dysfunction, need for reoperation to replace the prosthesis, immediate maternal death up to 42 days after delivery and late maternal death (up to 12 months after delivery). Obstetric complications included spontaneous abortion (interrupted pregnancy ≤ 22 weeks), premature delivery (gestational age ≤37 weeks), preeclampsia and postpartum hemorrhage. Newborn complications included neonatal death, prematurity, malformations related to warfarin embryopathy and the incidence of congenital heart disease.

This study was approved by the Research Ethics Committee SDC 4563/17/063 of the Hospital das Clinicas da Faculdade de Medicina da Universidade de São Paulo.

### Statistical analysis

The calculations were performed with the aid of the software R 4.2.0,^6^ and the graphs were built with the support of ggplot2.^7^ The descriptive analysis for quantitative variables is presented as means and standard deviations (mean ± standard deviation) or medians and interquartile ranges (median [Q1; Q3]) when the variables do not have a normal distribution using the Kolmogorov-Smirnov test; for qualitative variables, absolute and relative frequencies were considered. Fisher’s or Chi-square tests were used to test the association between the (qualitative) variables, when the variables have more than two categories. The t-test was used to compare quantitative variables when they had a normal distribution or the Wilcoxon-Mann-Whitney test (when the distribution of the variable was not normal, verified by the Kolmogorov-Smirnov test). A 5% significance level was adopted for the statistical tests and all were considered two-tailed.

The binary logistic regression model (occurrence or non-occurrence of at least one of the mentioned events) was adjusted in order to jointly verify the variables associated with the occurrence of cardiac complications, prosthesis dysfunction, intervention and/or death during and/or after pregnancy. Initially, variables with a p-value ≤ 0.15 in the association test with the outcome were selected through univariate analysis.

The univariate analysis was applied to an initial selection of variables with a p-value ≤ 0.15 in the association test with the outcome. The occurrence of any cardiac event and/or prosthesis dysfunction prior to pregnancy was also considered as a grouped variable. The selection of the best subset of variables (with the best fit) was based on the application of the stepwise method, using the Akaike criterion.^8^

## Results

**Comparative analysis of baseline characteristics between groups**: The mean age was higher in the BP group (p = 0.021), the time elapsed between implantation of the valve prosthesis and pregnancy was longer (p < 0.001) in the MP group; there was a difference in the distribution by etiology, with a higher prevalence of rheumatic etiology in the BP group (75.6% versus 50.0%, p < 0.001); cardiac events and/or prosthesis dysfunction were more frequent in the BP group (p < 0.001) (Table 1).

**Table 1:** – Maternal baseline characteristics

Measure	Total (n = 128)	Biological Prosthesis (n = 78)	Mechanical Prosthesis (n = 50)	p-value
**Patient's age (years)**	30.3 ± 6.2	31.3 ± 5.4	28.6 ± 7.0	0.021
**Valve Anatomical Position prosthesis**				0.329
Mitral	74/128 (57.8%)	45/78 (57.7%)	29/50 (58.0%)	
Aortic	34/128 (26.6%)	20/78 (25.6%)	14/50 (28.0%)	
Mitral + Aortic	14/128 (10.9%)	7/78 (9.0%)	7/50 (14.0%)	
Pulmonary	3/128 (2.3%)	3/78 (3.8%)	0/50 (0.0%)	
Tricuspid	3/128 (2.3%)	3/78 (3.8%)	0/50 (0.0%)	
ΔT Prosthesis implantation to Pregnancy	7.3 ± 6.4	5.5 ± 4.1	10.2 ± 8.2	<0.001
**Etiology of valve disease**				< 0.001
Rheumatic disease	84/128 (65.6%)	59/78 (75.6%)	25/50 (50.0%)	
Congenital heart disease	39/128 (30.5%)	14/78 (17.9%)	25/50 (50.0%)	
Infective endocarditis	5/128 (3.9%)	5/78 (6.4%)	0/50 (0.0%)	
**Cardiac event prior to pregnancy**				
Heart failure	14/128 (10.8%)	11/78 (14.1%)	3/50 (6.1%)	0.245
Thromboembolic Accident	9/128 (7.0%)	6/78 (7.7%)	3/50 (6.0%)	> 0.999
Atrial fibrillation	35/128 (27.3%)	26/78 (33.3%)	9/50 (18.0%)	0.067
Infectious endocarditis	15/128 (11.7%)	14/78 (17.9%)	1/50 (2.0%)	0.005
**Any cardiac event prior to gestation**	59/128 (46.1%)	45/78 (57.7%)	14/50 (28.0%)	0.001
**Prosthesis Function**				< 0.001
with dysfunction	21/128 (16.4%)	21/78 (26.9%)	0/50 (0.0%)	
without dysfunction	107/128 (83.6%)	57/78 (73.1%)	50/50 (100.0%)	
**Some cardiac event/dysfunction of the prosthesis prior to gestation**	66/128 (51.6%)	52/78 (66.7%)	14/50 (28.0%)	< 0.001
**Left ventricular dysfunction**				0.480
No	105/128 (82.0%)	62/78 (79.5%)	43/50 (86.0%)	
Yes	23/128 (18.0%)	16/78 (20.5%)	7/50 (14.0%)	

Δ T: time elapsed between the implantation of the prosthesis and the pregnancy under study.

**Analysis of clinical events and/or prosthesis dysfunction prior to pregnancy between groups:** Pregnancy success was achieved in 64 (50.0%) patients among whom 29 had no cardiac events and/or dysfunction before pregnancy, and there was no difference between groups (BP 56.4% versus MP 40.0% - p = 0.103) (Table 2).

**Table 2:** – Maternal-fetal evolution during pregnancy and 12 months postpartum

Measure	Total (n = 128)	Biological Prosthesis (n = 78)	Mechanical prosthesis (n = 50)	p-value
**Outcomes**				
**Pregnancy success**^**(1)**^	64/128 (50.0%)	44/78 (56.4%)	20/50 (40.0%)	0.103
Cardiac Complications or maternal death	55/128 (43.0%)	41/78 (52.6%)	14/50 (28.0%)	0.007
**Cardiac complications/ previous Dysfunction prosthesis/ during /after pregnancy**	46/128 (35.9%)	37/78 (47.4%)	9/50 (18.0%)	< 0.001
**Evolution of pregnancy up to 42 days postpartum**				
Heart failure	21/128 (16.4%)	17/78 (21.8%)	4/50 (8.0%)	0.050
Thromboembolism	3/128 (2.3%)	1/78 (1.3%)	2/50 (4.0%)	0.560
Atrial fibrillation	29/128 (22.7%)	24/78 (30.8%)	5/50 (10.0%)	0.009
Infective endocarditis	1/128 (0.8%)	1/78 (1.3%)	0/50 (0.0%)	> 0.999
At least one cardiac event (among the four above)	42/128 (32.8%)	34/78 (43.6%)	8/50 (16.0%)	0.001
Use of cardiovascular medication	57/128 (44.5%)	44/78 (56.4%)	13/50 (26.0%)	< 0.001
Hospitalization	33/128 (25.7%)	19/78 (24.4%)	14/50 (28.0%)	0.682
Maternal death	5/128 (3.9%)	3/78 (3.8%)	2/49 (4.0%)	> 0.999
**Obstetric complications**	43/128 (33.6%)	22/78 (28.2%)	21/50 (42.0%)	0.127
Type of delivery				0.191
Vaginal	31/107 (29.0%)	17/69 (24.6%)	14/38 (36.8%)	
Cesarean section	76/107 (71.0%)	52/69 (75.4%)	24/38 (63.2%)	
Fetal losses	23/128 (18.0%)	11/78 (14.1%)	12/50 (24.0%)	0.165
Weight of live NB (grams)	2748 ± 528 (n = 103)	2771 ± 576 (n = 65)	2708 ± 437 (n = 38)	0.530
Gestational age of delivery	37.0 [36.0; 38.0] (n = 111)	37.0 [37.0; 37.0] (n = 73)	37.0 [36.0; 38.0] (n = 38)	0.860
**Complications of newborn**	29/105 (27.6%)	18/67 (26.9%)	11/38 (28.9%)	0.824
**Outcome 12 months postpartum/abortion (disregarding the 5 deaths)**				
Heart failure	22/123 (17.9%)	16/75 (21.3%)	6/48 (12.5%)	0.238
Thromboembolism	3/123 (2.4%)	0/75 (0.0%)	3/48 (6.2%)	0.057
Atrial fibrillation	16/123 (13.0%)	12/75 (16.0%)	4/48 (8.3%)	0.278
Infectious endocarditis	1/123 (0.8%)	1/75 (1.3%)	0/48 (0.0%)	> 0.999
At least one cardiac event (out of the four above)	33/123 (26.8%)	24/75 (32.0%)	9/48 (18.8%)	0.144
**Prosthesis Function – 12 months postpartum/abortion (disregarding 5 deaths)**				0.003
with dysfunction	21/123 (17.1%)	19/75 (25.3%)	2/48 (4.2%)	
no dysfunction		56/75 (74.7%)	46/48 (95.8%)	

(1) absence of: obstetric complications, NB complications, death, cardiac events or dysfunction for those who had no events prior to pregnancy.

There were 55 (43.0%) cases of patients with cardiac complications and/or maternal deaths, and 46 (35.9%) were recorded in patients who started pregnancy with previous events and/or prosthesis dysfunction, proportionally higher in the BP group (p < 0.001) (Table 2). Five maternal deaths occurred in patients with previous cardiac events and concomitant obstetric and/or fetal complications.

**Analysis of cardiac complications/maternal deaths in the BP group during pregnancy and 12 months postpartum.** Seventeen (21.8%) patients experience HF during pregnancy, nine had a history of HF previous to gestation. HF were associated with permanent AF, ventricular dysfunction and valve prosthesis dysfunction. There was one (1.3%) case of coronary embolism in a patient with permanent AF, using LMWH, at the 16th week of pregnancy, which evolved into spontaneous abortion. Another (1.3%) patient presented with infective endocarditis at the 22nd week of pregnancy, by agent of the HACEK group (H.parainfluenza), evolved with septic shock, and obtained a good response to conventional treatment, and reached cesarean section at 37 weeks, uneventful and healthy newborn. Three maternal deaths associated with cardiogenic shock were recorded in patients with calcified bioprosthesis and severe stenosis, two during pregnancy and another in the immediate postpartum period. Follow-up over 12 months postpartum, there was a case of infectious endocarditis 3 months after delivery by Coxiella Bunetti, which had good clinical evolution with conventional treatment, but evolved with prosthesis dysfunction.

**Structural analysis of the prosthesis in the BP group**. Twenty-one (26.9%) patients started pregnancy with dysfunction of the biological prosthesis, nine of whom had stenosis with calcification and had poor clinical outcomes, including two maternal deaths, two emergency heart surgeries for valve replacement and one valve-in-valve procedure. There were six cases (6/57 – 10.5%) of “new” prosthesis dysfunction over 12 months postpartum.

**Analysis of cardiac complications and deaths in the MP group during pregnancy and 12 months postpartum.** Four (8.0%) patients had HF during pregnancy, of which three had significant ventricular dysfunction and two had HF before pregnancy. There were two (4.0%) cases of thromboembolism, both with LMWH, and two maternal deaths, one due to prosthesis thrombosis and the other due to HF in a patient with ventricular dysfunction. In the 12 months postpartum, there were three (6.2%) cases of thromboembolism, two of them with prosthesis thrombosis, one of which progressed to death.

**Structural analysis of the prosthesis in the MP group.** There were three cases of MP dysfunction caused by prosthesis thrombosis, one case during pregnancy that progressed to maternal death, and two other cases, on the 48th and 64th day after delivery respectively, requiring emergency cardiac surgery.

**Analysis of obstetric and fetal complications in the BP and MP groups.** The incidence of obstetric complications and in live newborns was not different between groups (Table 2). In the BP group, nine (11.5%) spontaneous abortions, four (5.1%) cases of pre-eclampsia, 19 (24.4%) premature births and three (6.4%) cases of postpartum hemorrhage were recorded. Besides abortions, there were two stillbirths and two deaths in the neonatal period. Among the live newborns, three presented malformations that corresponded to 1) mild cerebral palsy (mother undergoing valve replacement during pregnancy); 2) interventricular communication and renal agenesis, and 3) ventricular communication. In the MP group, there were 12 (24%) spontaneous abortions, six (12%) preterm deliveries and five (10%) cases of postpartum maternal hemorrhage. Of the 12 abortions, seven were in patients with LMWH and four in patients with sodium warfarin. Of the 38 live newborns, four (10.5%) had congenital heart defects and another (2.6%) had cerebral intraparenchymal hemorrhage identified in the 29th week of pregnancy, while taking sodium warfarin.

**Analysis of maternal and fetal complications according to the use of anticoagulants:** Seventy-one (55.4%) patients used anticoagulants, 45 (63.4%) patients in the PG subgroup on a sequential schedule and 26 (36.6%) patients in the NP subgroup. (Table 4). There was a lower frequency of fetal losses (8.9% versus 46.2% - p < 0.001) in the PG subgroup (Table 4A). Twenty-two patients in the BP+AF group had a lower percentage, although not significant, of pregnancy success compared to the BP group (Table 4B)

**Table 4:** – Proportion of Patients and Adherence to Anticoagulation Regimens

	Total (n = 128)	Prosthesis biological (n = 78)	Prosthesis mechanical (n = 50)	p-value
**Anticoagulant - yes**	71/128 (55.4%)	22/78 (28.2%)	49/50 (98.0%)	< 0.001
**Scheme used**				
Sequential protocol (PG subgroup)	45/71 (63.4%)	12/22 (54.5%)	33/49 (67.3%)	0.003
LMWH during pregnancy (NP subgroup)	11/71 (15.5%)	8/22 (36.4%)	3/49 (6.1%)	
Warfarin during pregnancy (NP subgroup)	15/71 (21.1%)	2/22 (9.1%)	13/49 (26.5%)	

The univariate analysis selected the type of prosthesis (biological or mechanical) (odds ratio 0.35; CI 95% [0.16; 0.74]; p = 0.007), Δ T prosthesis implantation (odds ratio 0.94; CI 95% [0.88; 0.99]; p = 0.045), etiology of valve disease (rheumatic or other) (odds ratio 1.8; CI 95% [0.8; 3.8]; p = 0.144), pre-existing HF at pregnancy (odds ratio 9.9; CI 95% [2.6; 65.6]; p = 0.004), AF (odds ratio 12.5; CI 95% [4.9; 36.5]; p < 0.001), prosthesis dysfunction (odds ratio 11.4; CI 95% [3.6; 50.7]; p < 0.001), left ventricular dysfunction (odds ratio 2.4; CI 95% [0.98; 6.3]; p = 0.06), and the use of anticoagulants (odds ratio 2.1; CI 95% [1.0; 4.3]; p = 0.05), as predictive variables of cardiac complications and maternal death (Table 3 - Figure 2A). Multivariate analysis showed that in the joint model, pre-existing HF (odds ratio 8.5; CI 95% [1.4; 50.7]; p = 0.019), AF (odds ratio 16.7; CI 95% [5.7; 49.1]; p < 0.001) and biological prosthesis dysfunction (odds ratio 12.6; CI 95% [3.0; 52.7]; p = 0.001) were the variables with the greatest predictive power for maternal complications and/or deaths (Table 5 - Figure 2B).

**Table 3:** – Cardiac complications and maternal death according to baseline characteristics before pregnancy

Measure	Total (n = 128)	Maternal complication or death		p-value
		No (n = 73)	Yes (n = 55)	
**Prosthesis**				0.007
biological	78/128 (60.9%)	37/73 (50.7%)	41/55 (74.5%)	
mechanical	50/128 (39.1%)	36/73 (49.3%)	14/55 (25.5%)	
**Patient's age (years)**	30.3 ± 6.2	29.9 ± 6.7	30.8 ± 5.4	0.156
**Prosthesis Anatomical Position prosthesis**				0.157
Mitral	74/128 (57.8%)	37/73 (50.7%)	37/55 (67.3%)	
Aortic	34/128 (26.6%)	21/73 (28.8%)	13/55 (23.6%)	
Mitral + Aortic	14/128 (10.9%)	9/73 (12.3%)	5/55 (9.1%)	
Pulmonary	3/128 (2.3%)	3/73 (4.1%)	0/55 (0.0%)	
Tricuspid	3/128 (2.3%)	3/73 (4.1%)	0/55 (0.0%)	
ΔT Prosthesis Implantation-Pregnancy	7.3 ± 6.4	8.3 ± 6.7	6.0 ± 5.8	0.051
**Etiology of valve disease**				0.089
Rheumatic disease	84/128 (65.6%)	44/73 (60.3%)	40/55 (72.7%)	
Congenital heart disease	39/128 (30.5%)	24/73 (32.9%)	15/55 (27.3%)	
Infective endocarditis	5/128 (3.9%)	5/73 (6.8%)	0/55 (0.0%)	
**Cardiac event prior to pregnancy**				
Heart failure	14/128 (10.9%)	2/73 (2.7%)	12/55 (21.8%)	< 0.001
Thromboembolic Accident	9/128 (7.0%)	3/73 (4.1%)	6/55 (10.9%)	0.171
Atrial fibrillation	35/128 (27.3%)	6/73 (8.2%)	29/55 (52.7%)	< 0.001
Infective endocarditis	15/128 (11.7%)	10/73 (13.7%)	5/55 (9.1%)	0.581
**Prosthesis Function**				< 0.001
with dysfunction	21/128 (16.4%)	3/73 (4.1%)	18/55 (32.7%)	
without dysfunction	107/128 (83.6%)	70/73 (95.9%)	37/55 (67.3%)	
**Any cardiac event/prosthesis dysfunction prior to pregnancy**	66/128 (51.6%)	20/73 (27.4%)	46/55 (83.6%)	< 0.001
**Left ventricular dysfunction**				0.066
No	105/128 (82.0%)	64/73 (87.7%)	41/55 (74.5%)	
Yes	23/128 (18.0%)	9/73 (12.3%)	14/55 (25.5%)	
**Use of anticoagulant**				0.072
No	57/128 (44.5%)	38/73 (52.1%)	19/55 (34.5%)	
Yes	71/128 (55.5%)	35/73 (47.9%)	36/55 (65.5%)	

Δ T: time elapsed between the implantation of the prosthesis and the pregnancy under study.

**Table 5:** – Result of the final model for maternal risks

Coefficient	estimate	standard error	Odds ratio (RC)	95% CI		p-value
Intercept	-1.6	0.3				< 0.001
Heart failure - yes	2.1	0.9	8.5	1.4	50.7	0.019
Atrial fibrillation - yes	2.8	0.6	16.7	5.7	49.1	< 0.001
Prosthesis Function - with dysfunction	2.5	0.7	12.6	3.0	52.7	0.001

## Discussion

The present study, which analyzed 128 pregnant women with valve prostheses, showed that the rate of full-term pregnancies, without cardiac, obstetric and/or fetal complications, did not exceed half of the cases studied.

These results reinforce the concept in the world literature that women with valve prostheses, whatever they may be, have a low success rate in pregnancy,^2,3,5,9^ which can be attributed to factors such as natural complications of valve disease, structural dysfunction of the prosthesis and risks related to the use of anticoagulants.

The highlight of this cohort was the proportion of 65% of cases of rheumatic etiology, indicative of a persistent reality in Brazil, where the occurrence of valve disease in young women has a strong association with rheumatic disease.

It is worth remembering that the cardiocirculatory overload and hypercoagulable state, physiological in pregnancy and the puerperium,^10,11^ are the main determinants of the occurrence of complications in women with prosthetic valves.

In this study, the standardization of the bovine pericardial biological prosthesis in 78 women and the double leaflet mechanics (St.Jude Medical) in the other 50 patients followed a protocol practice of the Institution, considering the clinical conditions at the time of surgery, the best results in terms of survival free of prosthesis dysfunction and the shared decision with the patient.^12-14^

**Clinical and structural evolution of the biological prosthesis (Group BP):** It was notable that the 75.6% prevalence of rheumatism in the BP group certainly justified the high frequency and combination of pre-existing cardiac events during pregnancy, such as AF and HF, sequelae attributable to the slow and insidious evolution of rheumatic pancarditis over decades.^15^

Among the characteristics of the BP group presented in Table 1, it is noted that the patients started pregnancy with an unfavorable clinical feature for the evolution of pregnancy, which led to a higher number of maternal complications in this group (p = 0.007 - Table 3). In fact, the present study also demonstrated that pre-existing cardiac events were predictors of cardiac complications and maternal death during pregnancy and after childbirth (Table 5 - Figure 2).

**Figure 2:** – Logistic Regression Analysis of Univariate (Fig 2A) and Multivariate (Fig 2B) Models for Cardiac Complications and Maternal Mortality, Considering Pre-Pregnancy Variables.

The world literature highlights the poor maternal and fetal prognosis of pregnant women with heart disease living in emerging countries with low and medium socioeconomic and cultural levels of the population.^16,17^ In this scenario, the Indian Registry of Heart Disease and Pregnancy (M-Pac) showed a 42.1% prevalence of rheumatic valve disease among 1029 pregnant women with heart diseases, and documented a significantly higher percentage of maternal death (8.6%) and cardiac complications (34.3%) among patients with valve prostheses when compared to other structural cardiac lesions.^18^

However, the Dutch study by Lameijer et al.^19^that included 78 pregnant women with valve prostheses only of congenital etiology, showed 17% of cardiac complications with a predominance of mechanical prostheses, and drew attention to the dysfunction of biological prostheses pre-existing during pregnancy as a marker of high risk for pregnancy complications.

From a structural point of view, this study found that biological prostheses performed worse, given that at an average of five years after implantation, 27% of structural prosthesis dysfunctions identified at the first consultation during pregnancy were documented, which had a high significance in the predictive value of poor maternal outcome (Figure 2).

These results were in agreement with Wichert-Schmitt et al.,^20^ who recorded 27% of dysfunctions of biological prostheses at the beginning of pregnancy in a cohort of 125 patients, with a significant correlation between dysfunction and the worst maternal-fetal evolution, particularly with prostheses located to the left of the heart.

In addition, the crucial point of the present study was the serious consequences of calcified dysfunctions in biological prostheses, which resulted in three maternal deaths resulting from cardiogenic shock. Given these results, current expert opinion is in favor of reoperation surgery to replace the biological prosthesis, when calcified, either during pregnancy, but ideally when planning a “new” pregnancy, even in asymptomatic patients.^21^

This study also brought up a controversial discussion by highlighting five cases of “new” prosthesis dysfunction over 12 months postpartum, one of which was caused by infective endocarditis three months after delivery. This result should be attributed to the natural survival of the biological prosthesis over the years, since it has already been shown that pregnancy does not influence the durability of biological prostheses.^22-24^

**Clinical and structural evolution of mechanical prosthesis (MP Group):** On the other hand, the patients in the MP group were younger, 50% of them had congenital etiology, and despite the longer time elapsed since the implantation of the prosthesis, these patients started the pregnancy with fewer complicating factors, which in principle indicated a more favorable clinical course (Table 3).

However, despite the absence of previous mechanical prosthesis dysfunction and the more favorable clinical characteristics at the beginning of pregnancy, only 40% achieved a successful pregnancy, recalling the three cases of prosthesis thrombosis, one in the first trimester of pregnancy with a fatal outcome, and two after delivery, requiring emergency surgery. The complexity of this situation reinforces the importance of supervision that is not limited to pregnancy, but should extend to the postpartum period.

It is estimated that 5 to 20% of patients have thromboembolic and/or hemorrhagic complications during pregnancy, childbirth or puerperium, due to the use of anticoagulants.^25-27^ The European Cardiac and Pregnancy Registry of the European Society of Cardiology-ROPAC, which included 212 pregnant women with mechanical prostheses, showed that only 58% of them had an uncomplicated pregnancy, and prosthesis thrombosis occurred in 4.7% (95% CI, 3.06-7.26) of cases with an 18% rate of maternal death.^28^

In view of this, the Statement of Pregnancy and Heart Disease of the Brazilian Society of Cardiology^21^ and the World Health Organization classify patients with mechanical prostheses at high risk for pregnancy (Class III- OMSm)^29^ and recommend that these pregnancies should be monitored in a tertiary hospital with a specialized multidisciplinary team.

**Protocol for the use of anticoagulants**: Guidance on the use of anticoagulants in pregnancy involves weighing up the risks of thrombosis and maternal bleeding, and the teratogenic and hemorrhagic risks to the fetus. Currently, there is no position on the best anticoagulant regimen for pregnant women with heart disease, since no pharmacological option, alone or in combination, provides evidence of efficacy in not causing adverse effects to the mother and/or fetus.

The protocol adopted in this study was supported by the scientific literature, validated by the best results and based on the experience of the Institution.^30,31^ From the point of view of bioethics, there was a constant concern to maintain the maternal benefit of anticoagulants, and avoid the harm of their teratogenic effects.

In this sense, clinical studies defend the efficacy of sodium warfarin in preventing thromboembolism in mechanical prostheses or valve disease with AF and, at the same time, discourage the use of LMWH given its association with a high incidence of thromboembolic events and maternal death.^25-27^

In fact, this study recorded 6.5% of cases of prosthesis thrombosis with the use of LMWH, despite the adjustment of doses according to plasma levels of between 1.0 and 1.2 IU/ml of anti-Xa factor activity. In practice, there are serious difficulties in achieving therapeutic goals with LMWH, since anti-Xa factor levels fluctuate significantly over 24 hours.^32^

The prospective study that included 15 pregnant women using therapeutic doses of enoxaeparin showed that the mean levels of anti-factor Xa activity were sub-therapeutic in 73% (11/15) of the cases, a condition that may explain the thrombotic events associated with LMWH.^33^

It is worth mentioning that the guidance that the patients received in the post-operative period of the prosthesis implant, regarding the risks of possible complications and the recommended regimen in the event of pregnancy, was the differential in this study. Proper pre-conception counseling, including guidance on contraception, is not the reality, it is estimated that only 5% of women with heart valve prostheses are oriented to the use of contraceptive methods and that about 5% to 10% of women are aware of the diagnosis of their heart disease.^1,16,28^

Even with the previous guidance obtained in our study, 26 (36.6%) of the patients did not use the recommended sequential scheme, either due to the event of unplanned pregnancy or personal decisions, and ended up suffering a greater number of spontaneous abortions (Table 4 A). Interestingly, cardiac complications and maternal deaths were not higher among patients who used anticoagulants (Table 3). However, fetal losses and cardiac complications among these patients were higher in the NP subgroup and BP=AF group, respectively (Table 4A and 4B).

**Table 4-A:** – A Analysis of maternal and fetal complications in PG subgroups versus NP

	Total with anticoagulation	Anticoagulation scheme		p-value
		PG (n = 45)	NP (n = 26)	
**Prosthesis**				0,425
Biological	22/71 (31.0%)	12/45 (26.7%)	10/26 (38.5%)	
Mechanical	49/71 (69.0%)	33/45 (73.3%)	16/26 (61.5%)	
**Maternal complications (including death)**	36/71 (50.7%)	21/45 (46.7%)	15/26 (57.7%)	0.462
**Fetal losses**	16/71 (22.5%)	4/45 (8.9%)	12/26 (46.2%)	< 0.001
**Newborn complications**	17/55 (30.9%)	12/41 (29.3%)	5/14 (35.7%)	0.742
**Pregnancy success**	30/71 (42.3%)	22/45 (48.9%)	8/26 (30.8%)	0.212

**Table 4-B:** – Analisys of matenal and fetal complications of BP group vs BP+AF subgroup

	Total PB (n = 78)	Using anticoagulant		p-value
		No (n = 56)	Yes (n = 22)	
Maternal complications (including death)	41/78 (52,6%)	19/56 (33,9%)	22/22 (100,0%)	< 0,001
Fetal losses	11/78 (14,1%)	6/56 (10,7%)	5/22 (22,7%)	0,276
Newborn complications	18/67 (26,9%)	12/50 (24,0%)	6/17 (35,3%)	0,364
Pregnancy success	44/78 (56,4%)	34/56 (60,7%)	10/22 (45,5%)	0,311

Sequential protocol – LMWH in the first trimester, sodium warfarin, from the second trimester to the 36th week of pregnancy; PG Group – planned pregnancy; NP Group – did not plan pregnancy ; LMWH – low molecular weight heparin, group BP – biological prosthesis without anticoagulant group BP+AF biological prosthesis associated with atrial fibrillation using anticoagulant.

**Obstetric and fetal complications:** It is likely that a high incidence of obstetric and fetal complications, especially in the group that did not follow the sequential scheme (Table 4B), is the consequence of the use of sodium warfarin, which can cause spontaneous abortions in about 10% to 30% of cases.^34^ In addition, sodium warfarin is mentioned as teratogenic and causes malformations known as “fetal warfarin syndrome,” regardless of dose, however, it has not been identified in any case in this study.^35-37^

Another complication, fetal intracranial hemorrhage, is uncommon, usually occurring in the second trimester of pregnancy and in the therapeutic ranges of anticoagulation with sodium warfarin.^38^ Although fatal in most cases, these hemorrhagic accidents can cause severe sequelae in surviving newborns, as occurred in the present study. It is worth remembering that the pharmacokinetics of sodium warfarin results in a high blood concentration in the fetus, which cannot be accurately estimated in maternal blood tests.^39^

Another fact that drew attention was the identification of 5.8% of congenital heart diseases in live newborns, a rate five times higher than the prevalence of 0.8% to 1.0% in the general population.^40^ Perhaps, the hereditary character of congenital heart diseases present in this series contributed to this high incidence, in addition to the use of sodium warfarin that cannot be ruled out.

It is also worth mentioning the occurrence of a case of neurological impairment of the fetus resulting from cardiac surgery during the second trimester of pregnancy. This serious complication, already described in the literature, is hypothesized to be the neurological sequelae caused by intra-uterus in response to laminar and non-pulsatile flow, hemodilution, and hypothermia used in cardiopulmonary bypass.^41^

## Final considerations

The number of participants in this study was not large, and because it was conducted in a single reference center in cardiology, it is not exempt from selection influences on patients. However, it is possible to highlight differences in methodology that highlight the quality and scope of this research, such as: 1) follow-up of patients since implantation of the valve prosthesis; 2) reproductive counseling before pregnancy; 3) guidance on the anticoagulant regimen to be followed at the beginning of pregnancy; 4) standardization of prostheses in the consistency of results; 5) follow-up of patients up to 12 months after delivery, a period considered late maternal death, and 6) study period was limited to five years to avoid changes in clinical practice over time.

## Conclusions

Patients with valve prostheses face a low success rate in pregnancy. The complicating factors associated with valve disease, the limited durability of bioprostheses, and the challenges of managing anticoagulation during pregnancy are significant barriers to maternal and fetal success. The choice of prosthesis for women considering future pregnancies should not be made in isolation but rather within the broader context of the complex progression of heart disease. The low success rates observed in this study underscore the critical importance of meticulous pregnancy planning and highlight the need for further research focused on women of reproductive age with valve disease.
